# The Relationship between the complement system and subclinical carotid atherosclerosis in patients with rheumatoid arthritis

**DOI:** 10.1186/s13075-024-03360-3

**Published:** 2024-07-08

**Authors:** Marta Hernández-Díaz, Dara Rodríguez-González, Elena Heras-Recuero, Fuensanta Gómez-Bernal, Juan Carlos Quevedo-Abeledo, Agustín F. González-Rivero, Elena González-López, J. Gonzalo Ocejo-Vinyals, Alejandro Jimenez-Sosa, Miguel Ángel González-Gay, Iván Ferraz-Amaro

**Affiliations:** 1 Division of Rheumatology, Hospital Universitario de Canarias, Tenerife, Santander, Spain; 2 Division of Central Laboratory, Hospital Universitario de Canarias, Tenerife, Spain; 3 Division of Rheumatology, IIS-Fundación Jiménez Díaz, Av. de los Reyes Católicos, 2, Madrid, 28040 Spain; 4 Division of Rheumatology, Hospital Doctor Negrín, Las Palmas de Gran Canaria, Spain; 5 Division of Immunology, Hospital Universitario Marqués de Valdecilla, Santander, Spain; 6 Research Unit, Hospital Universitario de Canarias, Tenerife, Spain; 7 Department of Medicine and Psychiatry, University of Cantabria, Santander, Spain; 8 Department of Internal Medicine, University of La Laguna (ULL), Tenerife, Spain

**Keywords:** Rheumatoid arthritis, Complement system, Carotid plaque, Intima media thickness, Atherosclerosis, Cardiovascular disease

## Abstract

**Background:**

Patients with rheumatoid arthritis (RA) have an increased risk of cardiovascular (CV) events and CV mortality. Subclinical carotid atherosclerosis is independently associated with rates of incident CV events among patients with RA. The complement system has been related to both the etiopathogenesis of RA and CV disease. In this study, we aimed to evaluate the association between a comprehensive assessment of the complement system and carotid intima media thickness and carotid plaque in patients with RA.

**Methods:**

430 patients with RA were recruited. Functional assays of the three pathways of the complement system, utilizing new-generation techniques, were assessed. Additionally, serum levels of individual components of the complement system belonging to the three pathways were measured: C1q (classical), lectin (lectin), C2, C4, and C4b (classical and lectin), factor D and properdin (alternative), C3 and C3a (common), C5, C5a, and C9 (terminal), as well as regulators factor I and C1-inhibitor. Subclinical carotid atherosclerosis was evaluated by ultrasonography. Multivariable linear regression analysis was conducted to investigate the association between the complement system and carotid intima media thickness and carotid plaque.

**Results:**

After multivariable adjustment, which included traditional CV risk factors and disease-related data, C3a and C5a exhibited significant positive correlations with carotid intima media thickness. Additionally, higher values of C1-inhibitor, properdin, C3, C5, and C5a were independently associated with the presence of carotid plaque.

**Conclusion:**

The complement system and subclinical carotid atherosclerosis are linked in patients with RA.

**Supplementary Information:**

The online version contains supplementary material available at 10.1186/s13075-024-03360-3.

## Background

Rheumatoid arthritis (RA) is an inflammatory peripheral polyarthritis characterized by symmetric joint involvement. It often results in joint damage by eroding cartilage and bone. Additionally, individuals with RA have a higher incidence of atherosclerosis compared to those without the condition [1], resulting in an elevated incidence of cardiovascular (CV) events such as stroke, myocardial infarctions, and cardiac deaths when compared to the general population [2, 3]. It is known that in addition to the traditional CV risk factors, the presence of chronic inflammation can explain the development of accelerated atherosclerosis in these patients [4] through effects mediated by cytokines, immune complexes and endothelial dysfunction, or by a combination of these factors [1, 5–7]. Besides, higher prevalence of carotid subclinical atherosclerosis has been described in RA patients [8], and the presence of carotid plaques predicts the development of CV events and death in patients with RA [9].

The complement system plays a crucial role in the innate immune response, working alongside antibody-mediated processes. Comprising around 60 proteins found in both plasma and cell membranes, this system consists of three interconnected activation pathways: the classical, alternative, and lectin cascades. Additionally, there is a shared terminal lytic pathway and a complex network of regulators and receptors [10]. Each pathway is triggered by distinct mechanisms, yet all converge to activate C3 and deposit it as C3b, a central event in complement activation. The classical pathway is initiated by antibodies, whereas the lectin pathway rapidly identifies repetitive carbohydrate patterns on microbial pathogens’ surfaces. In contrast, the alternative pathway acts as an ancient surveillance system, representing the original extracellular complement pathway. It can be activated independently of antibodies or lectins and operates continuously at a low level, facilitated by the presence of a labile thioester bond at C3, a phenomenon known as “tick-over.”

The complement system has been linked to the etiopathogenesis of RA. In this sense, in individuals with RA, a diverse range of autoantibodies is present, some of which can bind to antigens found within the joints. This binding process leads to the formation of immune complexes within the inflamed synovial tissue (pannus) and cartilage, subsequently triggering complement activation and consumption of complement components [11]. Consequently, activation products such as C1 inhibitor-C1r-C1s complexes, C2a, C3a, C3d or C3dg, and C5a have been observed at elevated levels in synovial fluid [12], and complement deposition can be visualized in synovial tissue through immunohistochemical staining [13].

In this study, we conducted a comprehensive analysis of the complement system, including functional tests of its three pathways and the assessment of serum complement elements from both upstream and downstream of the system. Additionally, carotid ultrasound was performed to determine the presence of carotid plaque and carotid intima-media wall thickness (cIMT). Our study aimed to investigate the relationship between the complement system and the presence of subclinical carotid atherosclerosis in a large series of patients with RA, considering the presence of traditional CV risk factors.

## Materials and methods

### Study participants

Cross-sectional study that included 430 patients with RA recruited consecutively from 2019 to 2021. All participants were 18 years old or older and met the 2010 ACR/EULAR classification criteria [14]. They had been diagnosed by rheumatologists and were undergoing regular follow-up appointments at rheumatology outpatient clinics. To be included in the present study, participants were required to have a duration of RA disease of at least one year. As glucocorticoids are frequently utilized in RA treatment, patients receiving prednisone or an equivalent dose of ≤ 10 mg/day were eligible for participation in the study. Patients with a history of cancer, hypothyroidism, nephrotic syndrome, as well as those displaying evidence of active infection, were excluded from the study. Additionally, having had a previous CV event was an exclusion criterion for inclusion in this study. A flowchart illustrating the excluded and included patients is illustrated in Supplementary Fig. 1. The study protocol was approved by the Institutional Review Committee at Hospital Universitario de Canarias and at Hospital Universitario Doctor Negrín (both in Spain), and all subjects provided informed written consent (approval no. 2019-452-1). All research activities were carried out in strict compliance with applicable guidelines and regulations, and in accordance with the principles set forth in the Declaration of Helsinki.

### Data collection, laboratory assessments and carotid ultrasound evaluation

Participants enrolled in the study underwent a comprehensive examination, which included completing a questionnaire regarding CV risk factors and medication usage. A thorough physical examination was conducted, which involved measurements such as body-mass index (BMI) calculated as weight in kilograms divided by the square of the height in meters, abdominal circumference, and assessment of systolic and diastolic blood pressure under standardized conditions. Additionally, information regarding smoking, diabetes, and hypertension was gathered. Specific diagnoses and medication details were verified through a review of medical records. Cholesterol, triglycerides, and HDL-cholesterol were measured using the enzymatic colorimetric assay. LDL-cholesterol was calculated using the Friedewald formula. Dyslipidemia was defined if one of the following was present: total cholesterol > 200 mg/dL, triglycerides > 150 mg/dL, HDL cholesterol < 40 in men or < 50 mg/dL in women, or LDL cholesterol > 130 mg/dL. A standard technique was used to measure the erythrocyte sedimentation rate (ESR) and high-sensitivity C-reactive protein (CRP). Disease activity in patients with RA was measured using the Disease Activity Score (DAS28) in 28 joints [15] using both ESR (DAS28-ESR) and CRP (DAS28-CRP) in its calculation, the Clinical Disease Activity Index (CDAI) [16] and the Simple Disease Activity Index (SDAI) [17].

### Complement assessments

The SVAR functional complement assays under the Wieslab^®^ brand (Sweden) were used to assess classical, alternative and lectin pathways activity. These tests combine principles from the hemolytic assay for complement function with the use of labeled antibodies that specifically target the neoantigen produced as a result of complement activation. The quantity of neoantigen generated is directly proportional to the functional activity of the complement pathways. Microtiter strip wells are coated with classical, alternative or lectin pathway-specific activators. In this process, the patient’s serum is diluted with a specific blocker to ensure activation of only the studied complement pathway. During the incubation of the diluted patient serum in the wells, the specific coating activates C. Subsequently, the wells are washed, and the presence of C5b-9 is detected using an alkaline phosphatase-labeled specific antibody against the neoantigen expressed during membrane attack complex (MAC) formation. Following an additional washing step, specific antibodies are detected by incubating with an alkaline phosphatase substrate solution. The intensity of the color developed correlates with the amount of complement activation and is measured in terms of absorbance (optical density). The quantity of formed Membrane Attack Complex (MAC) neo-epitope reflects the activity of the complement cascade. The result is expressed semi-quantitatively by calculating the optical density ratio between a positive control and the sample. It is crucial to note that for the classical, alternative, and lectin cascade values, lower levels indicate a higher activation of the respective pathway. Wieslab^®^ has validated these functional assays by studying their correlation and concordance with the classical CH50 and AH50 hemolytic tests (https://www.svarlifescience.com/). Additionally, complement individual elements were assessed through MILLIPLEX^®^ map Multiplex Detection (MERCK^®^, Cat. No. HCMP1MAG-19 K and No. HCMP2MAG-19 K). To achieve a comprehensive characterization of all complement pathways, panels were devised to evaluate various components, including C1q (classical pathway), lectin (lectin pathway), C1 inhibitor, C2, C4, and C4b (classical and lectin pathways), factor D and properdin (alternative pathway), C3, C3a, and factor I (common pathway), as well as C5, C5a, and C9 (terminal pathway). Both intra- and inter-coefficients of variability for these assays were maintained below 10%.

### Carotid ultrasound assessment

Carotid ultrasound examination was used to assess cIMT in the common carotid artery and to detect focal plaques in the extracranial carotid tree in patients with RA [18]. A commercially available scanner, the EsaoteMylab 70 (Genoa, Italy), equipped with a 7–12 MHz linear transducer and using an automated software-guided radiofrequency technique —Quality Intima Media Thickness in real-time (QIMT, Esaote, Maastricht, Holland)— was used for this purpose. cIMT value represents the maximum thickness from 3 measurements on each side. As previously reported [18], based on the Mannheim consensus, plaque criteria in the accessible extracranial carotid tree (common carotid artery, bulb, and internal carotid artery) were defined as follows: a focal protrusion in the lumen measuring at least cIMT > 1.5 mm; a protrusion at least 50% greater than the surrounding cIMT; or arterial lumen encroaching > 0.5 mm [19].

### Statistical analysis

Demographic and clinical characteristics in patients with RA were described using means (standard deviation) or percentages for categorical variables. For non-normally distributed continuous variables, data were expressed as median and interquartile range (IQR). The association between subclinical atherosclerosis and circulating complement system molecules and pathways was analyzed through multivariable linear and logistic regression analysis using complement system routes and elements, and carotid ultrasounds, respectively, as the independent and dependent variable, while adjusting for covariates. For the construction of a heatmap of multivariable associations, standardized beta coefficients were calculated and plotted. Using standardized beta coefficients, rather than non-standardized ones, facilitates comparison between beta coefficients in multiple associations. Covariates for the multivariable regression analysis were selected from demographic and disease-related data that exhibited a relationship with cIMT or carotid plaque with a p-value below 0.20. All analyses were conducted with a 5% two-sided significance level using Stata software, version 17/BE (StataCorp, College Station, TX, USA). P-values < 0.05 were considered statistically significant. Heatmap graphs were generated using GraphPad Prism version 10, GraphPad Software, San Diego, California, USA.

## Results

### Demographic and disease-related data

This study included a total of 430 patients diagnosed with RA. Demographic- and disease-related characteristics of the participants are shown in Table 1. The study population had a mean age of 55 ± 10 years, with 81% of the participants being women. Traditional CV risk factors were prevalent. In this sense, 34% had hypertension, 13% were diabetic, and 22% were smoking at the time the study was performed. The median duration of the disease was 8 years (interquartile range, IQR, 4–15). At the time of the study, the mean values of CRP and ESR were 2.7 mg/l (IQR 1.3–6.1) and 18 mm/1st hour (IQR 7–32), respectively. Rheumatoid factor was positive in 72% of patients, and 65% were positive for anti-citrullinated protein autoantibodies (ACPA). The disease activity, as measured by DAS28-ESR, was 3.1 ± 1.4. According to this score, 40% of the patients met the criteria for remission, while 18% and 42% were categorized in the low and moderate/high disease activity groups, respectively. The DAS28-CRP had a value of 2.7 ± 1.1, and SDAI and CDAI were 12 (IQR 7–19) and 8 (IQR 4–14), respectively. 36% of the patients were undergoing treatment with prednisone, while 87% were receiving at least one conventional disease-modifying antirheumatic drug (DMARD) of any type, with methotrexate being the most commonly prescribed (73%). 19% of the patients were receiving anti-tumor necrosis factor therapies. Carotid plaque was present in 180 (42%) of the patients, and cIMT had a value of 696 ± 131 microns. The frequency of usage of other treatments and historical disease-related data can be found in Table 1.

**Table 1:** Demographics and disease related data in RA patients

	Rheumatoid arthritis
	(n = 430)
Age, years	55 ± 10
Female, n (%)	350 (81)
BMI, kg/m^2^	28 ± 5
Abdominal circumference, cm	97 ± 13
Hip circumference, cm	106 ± 11
Abdominal to hip ratio	0.92 ± 0.08
Cardiovascular risk factors, n (%)	
Current smoker	93 (22)
Obesity	137 (32)
Hypertension	148 (34)
Diabetes Mellitus	54 (13)
Dyslipidemia	324 (77)
Statins	139 (32)
Carotid plaque	180 (42)
Intima media thickness, microns	696 ± 131
Disease related data	
Disease duration, years	8 (4–15)
CRP at time of study, mg/l	2.7 (1.3–6.1)
ESR at time of study, mm/1st hour	18 (7–32)
Rheumatoid factor, n (%)	303 (72)
ACPA, n (%)	253 (65)
DAS28-ESR	3.13 ± 1.35
DAS28-PCR	2.73 ± 1.08
SDAI	12 (7–19)
CDAI	8 (4–14)
History of extraarticular manifestations, n (%)	38 (10)
Erosions, n (%)	166 (43)
Current drugs, n (%)	
Prednisone	156 (36)
Prednisone doses, mg/day	5 (3–5)
NSAIDs	194 (45)
DMARDs	373 (87)
Methotrexate	316 (73)
Leflunomide	94 (22)
Hydroxychloroquine	45 (18)
Salazopyrin	28 (7)
Anti TNF therapy	83 (19)
Tocilizumab	23 (5)
Rituximab	7 (2)
Abatacept	12 (3)
JAK inhibitors	20 (5)

Dyslipidemia was defined if one of the following was present: total cholesterol > 200 mg/dL, triglycerides > 150 mg/dL, HDL cholesterol < 40 in men or < 50 mg/dL in women, or LDL cholesterol > 130 mg/dL. CRP: C reactive protein; ACPA: Anti-citrullinated protein antibodies. NSAID: Nonsteroidal anti-inflammatory drugs; DMARD: disease-modifying antirheumatic drug. TNF: tumor necrosis factor, ESR: erythrocyte sedimentation rate; BMI: body mass index; DAS28: Disease Activity Score in 28 joints; CDAI: Clinical Disease Activity Index; SDAI: Simple Disease Activity Index

Functional complement assays of the classical, alternative and lectin pathways, and single complement components, C1q, C1-inhibitor, C2, C4, C4b, C3, C3a, C5, C5a, and C9, and factor D and I, properdin and lectin serum values are presented in Supplementary Table 1.

### Univariable and multivariable analysis of the relationship between complement system and subclinical carotid atherosclerosis

**Fig. 1:**
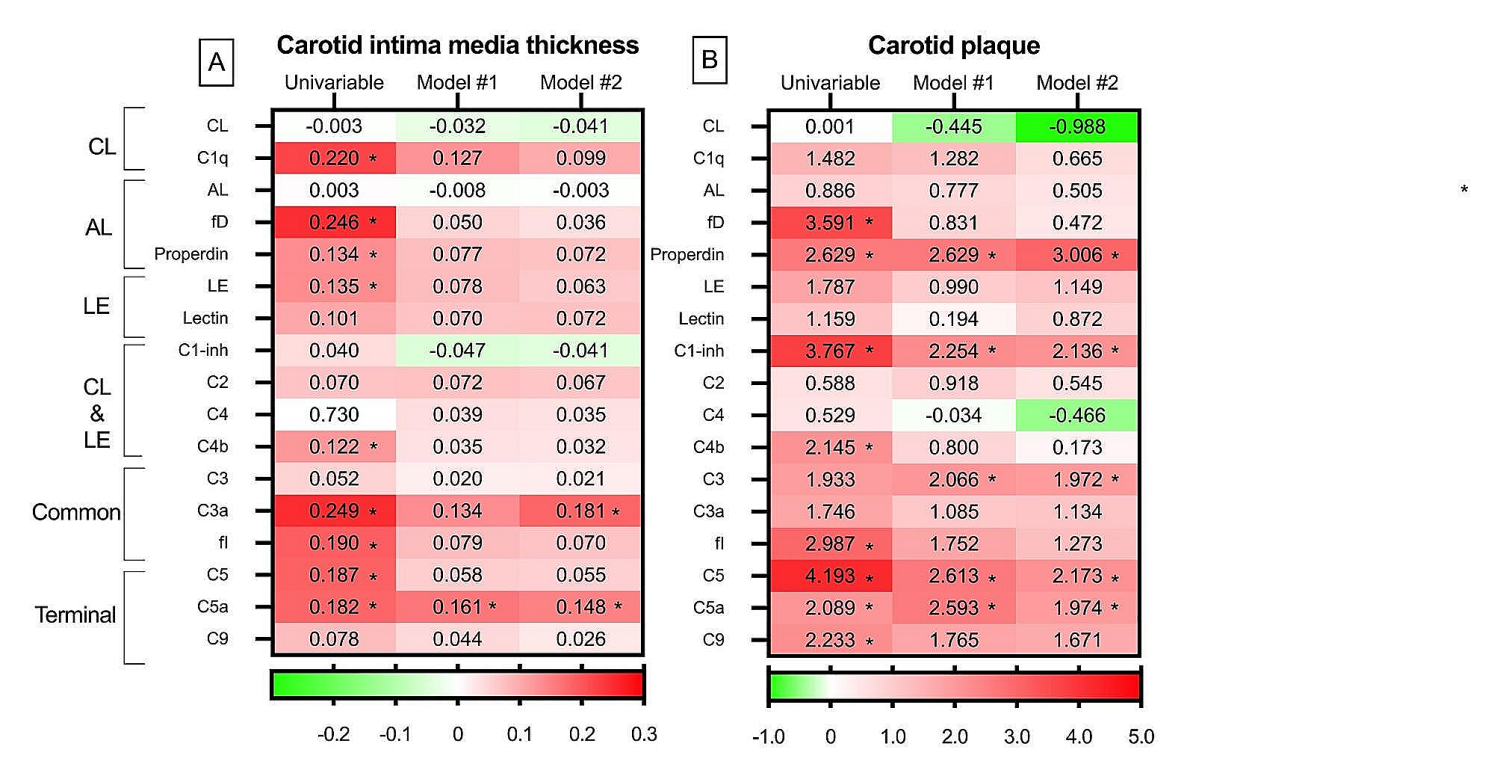
Heatmap of the relationship between complement system and carotid ultrasound assessment. Values in the cells represent multivariable standardized beta coefficients of the association between complement system (independent variable) and carotid intima media thickness **(A)** and carotid plaque **(B)** (dependent variables). Model 1 is adjusted for demographics and traditional cardiovascular factors, and Model 2 for Model 1 + disease related data. CL: classical, LE: lectin, AL: alternative, fI: factor I, fD: factor D. Significant correlation and standardized beta coefficients with a *p* < 0.05 are depicted as *

The relation of the complement system to carotid ultrasound evaluation is expressed in Fig. 1 as standardized beta coefficients, considering the complement system and carotid assessment as, respectively, the independent and dependent variables. Standardized beta coefficients provide a measure of the strength and direction of the relationship between variables when they are expressed in different units or scales. In this regard, standardizing the coefficients allows for comparisons by bringing all variables to a common scale.

Concerning cIMT, many positive associations (red in the heatmap) disclosed statistical significance in the univariable analysis. In this sense, C1q that represents an upstream molecule of the classical route, factor D and properdin of the alternative cascade, as well as C4b of the lectin and classical pathways, showed a positive and significant relation to cIMT. This was also the case for C3a and factor I of the common route, and C5 and C5a of the terminal cascade. After multivariable analysis that included demographics and traditional CV factors such as age, sex, abdominal circumference, hypertension, type 2 diabetes and the use of statins (Model #1 in Fig. 1-A), many significant associations were lost (red color decreased in the heatmap). Only C5a maintained a significant and positive relation to cIMT.

Due to potential influences from disease activity, medications used in treatment, and various other factors related to the disease, we conducted an additional multivariable linear regression analysis adjusting for rheumatoid factor and the use of NSAIDs, methotrexate, hydroxychloroquine and anti-TNF alpha therapies (Model #2 in Fig. 1-A). Remarkably, C3a and C5a continued to exhibit a positive and significant relation to cIMT after this full multivariable adjustment.

The analysis of the relation between complement system and carotid plaque yielded similar results to the ones of cIMT in the univariable analysis (Fig. 1-B). However, after adjustment for age, sex, abdominal circumference, hypertension, diabetes, the use of statins (Model #1 in Fig. 1-B), and disease duration, ESR, ACPA positivity and the utilization of NSAIDs and hydroxychloroquine (Model #2 in Fig. 1-B), a higher frequency of significant relations between the complement system and carotid plaque were found. In this regard, after this comprehensive adjustment, properdin of the alternative route, C1-inhibitor of the classical and lectin cascades, C3 of the common pathway, and C5 and C5a of the terminal, maintained a significant and positive association with the presence of carotid plaque (Fig. 1-B).

Additionally, non-standardized beta coefficients and odds ratios of the relation of complement system to cIMT and carotid plaque are presented in Table 2.

**Table 2:** Non-standardized beta coefficients of the relation of complement system to subclinical carotid atherosclerosis

	Carotid intima thickness, microns			Carotid plaque		
	Univariable	Model #1	Model #2	Univariable	Model #1*	Model #2*
	Beta coef. (95%) CI, p			OR (95%) CI, p		
**Classical pathway**						
Classical, %	-0.2 (-0.6-0.6), 0.95	-0.2 (-0.7-0.3), 0.47	-0.2 (-0.7-0.3), 0.37	1.00 (0.99–10.1) 0.99	0.99 (0.99-1.00), 0.66	0.99 (0.98–1.01), 0.32
C1q, mg/dl	**4 (0.8-7), 0.015**	2 (-0.4-5), 0.099	2 (-1-4), 0.22	1.04 (0.99–1.09), 0.14	1.04 (0.98–1.10), 0.20	1.02 (0.95–1.10), 0.51
**Alternative pathway**						
Alternative, %	0.02 (-0.5-06), 0.95	-0.04 (-0.5-0.4), 0.85	-0.02 (-0.5-0.5), 0.95	1.00 (0.99–1.01), 0.38	1.00 (0.99–1.01), 0.44	1.00 (0.99–1.01), 0.61
Factor D, mg/dl	**454 (272–637), < 0.001**	92 (-83-267), 0.30	66 (-117-250), 0.48	**273 (13-5853), < 0.001**	4.79 (0.12–193), 0.41	3.40 (0.02–549), 0.64
Properdin	**50 (12–87), 0.009**	29 (-4-62), 0.090	27 (-7-60), 0.12	**2.24 (1.23–4.08), 0.009**	**2.73 (1.29–5.76), 0.009**	**4.22 (1.65-11), 0.003**
**Lectin pathway**						
Lectin, %	**0.4 (0.09–0.7), 0.010**	0.2 (-0.02-0.5), 0.074	0.2 (-0.07-0.4), 0.16	1.00 (0.99–10.1), 0.074	1.00 (0.99–1.01), 0.32	1.00 (0.99–1.01), 0.25
Lectin, mg/dl	92 (-0.6-184), 0.052	64 (-15-144), 0.11	66 (-16-148), 0.11	2.32 (0.56–9.58), 0.25	1.18 (0.22–6.49), 0.85	2.56 (0.31-21), 0.38
**Classical and lectin pathways**						
C1-inh	0.8 (-1-3), 0.42	-0.9 (-2-0.7), 0.27	-0.8 (-2-0.9), 0.35	**1.06 (1.03–1.09), < 0.001**	**1.04 (1.00-1.07), 0.024**	**1.05 (1.00-1.09), 0.033**
C2, mg/dl	1 (-0.7-3), 0.23	1 (-0.4-3), 0.14	1 (-0.5-3), 0.19	1.01 (0.98–1.04), 0.56	1.02 (0.98–1.05), 0.36	1.01 (0.97–1.05), 0.59
C4, mg/dl	0.9 (-0.3-2), 0.14	0.5 (-0.6-2), 0.36	0.5 (-0.6-2), 0.42	1.01 (0.99–1.02), 0.60	0.99 (0.98–1.02), 0.97	0.99 (0.97–1.02), 0.64
C4b, mg/dl	**5 (0.8-9), 0.018**	1 (-2-5), 0.44	1 (-2-5), 0.50	**1.07 (1.01–1.14), 0.032**	1.02 (0.96–1.11), 0.42	1.01 (0.92–1.10), 0.86
**Common pathway**						
C3, mg/dl	0.2 (-0.2-0.7), 0.29	0.09 (-0.3-0.5), 0.66	0.01 (-0.3-0.5), 0.65	1.01 (0.99–1.01), 0.053	**1.01 (1.00-1.02), 0.039**	**1.01 (1.00-1.02), 0.049**
C3a, mg/dl	**3 (1–6), 0.006**	2 (-0.3-4), 0.085	**3 (0.3-5), 0.026**	1.04 (0.99–1.08), 0.081	1.03 (0.98–1.08), 0.28	1.03 (0.98–1.08), 0.26
Factor I, mg/dl	**21 (10–32), < 0.001**	9 (-1-19), 0.090	8 (-3-18), 0.14	**1.31 (1.10–1.57), 0.003**	1.22 (0.98–1.51), 0.080	1.20 (0.91–1.59), 0.20
**Terminal pathway**						
C5, mg/dl	**13 (6–20), < 0.001**	4 (-2-10), 0.21	4 (-3-10), 0.24	**1.36 (1.18–1.56), < 0.001**	**1.25 (1.06–1.47), 0.009**	**1.28 (1.02–1.60), 0.030**
C5a, mg/dl	**26 (12–40), < 0.001**	**23 (11–35), < 0.001**	**21 (8–34), 0.001**	**1.35 (1.02–1.79), 0.037**	**1.57 (1.12–2.21), 0.010**	**1.58 (1.00-2.48), 0.048**
C9, mg/dl	18 (-6-42), 0.13	10 (-10-30), 0.32	6 (-15-27), 0.57	**1.55 (1.05–2.27), 0.026**	1.50 (0.96–2.35), 0.078	1.73 (0.91–3.29), 0.095

In this analysis complement system is the independent variable and carotid intima media thickness and carotid plaque are the dependent variable

Model #1 adjusted for age, sex, abdominal circumference, hypertension, type 2 diabetes and the use of statins

Model #2 adjusted for Model #1 + rheumatoid factor and the use of NSAIDs, methotrexate, hydroxychloroquine and anti-TNF alpha therapies

Model #1* adjusted for age, sex, abdominal circumference, hypertension, type 2 diabetes and the use of statins

Model #2* adjusted for Model #1* + disease duration, ESR, ACPA positivity and the utilization of NSAIDs and hydroxychloroquine

## Discussion

Our study is the first to date to investigate the relationship between a comprehensive analysis of the complement system and subclinical carotid atherosclerosis in a large series of patients with RA. This has been performed including functionally assessment of three pathways of the complement system and by measuring the concentration of several upstream and downstream complement elements. Moreover, we have applied a fully multivariable adjustment that included traditional CV risk factors and disease-related data. Our results support a link between the complement system and the presence of subclinical carotid atherosclerosis in patients with RA and suggest a potential influence of complement in the development of atherosclerotic disease in these patients.

A relatively large body of data has been published regarding the role of the complement system in CV disease in general population [20]. This evidence comes from both prospective clinical studies and those based on genetic polymorphisms and anatomopathological findings. With respect to this, a study comprising 188 heart failure patients and 67 healthy controls matched for age and sex examined properdin, factor D, the alternative pathway inhibitor factor H, and the activation product, terminal complement complex [21]. Patients with heart failure had significantly increased levels of factor D and terminal complement complex, and decreased levels of properdin. Levels of factor D and properdin were correlated with measures of systemic inflammation, cardiac function, and deteriorated diastolic function. The authors concluded that dysregulation of circulating components of the alternative pathway explains the increased degree of complement activation, which is associated with disease severity in heart failure patients [21]. In another study that included a cohort of 389 men spanning a spectrum of risk who were referred for coronary angiography, a single baseline measurement of serum complement C4 level emerged as an independent predictor for the subsequent development of stroke [22]. This was also the case in a population-based prospective study of 5850 initially healthy men. In this study, both C3 and C4 displayed significant correlations with CV risk factors, such as blood pressure, BMI, and lipids. Elevated C4 levels were linked to CV disease incidence, regardless of traditional CV risk factors [23]. Other studies disclosed that high circulating levels of C3 and C3a are associated with an increased cIMT [24], peripheral artery disease [25], renal arteriolosclerosis in non-diabetic chronic kidney disease [26], and increased risk of myocardial infarction [27]. In RA, a disease associated increased cIMT and carotid plaques, which are surrogate markers of atherosclerotic CV disease [28], our findings indicate that the complement system appears to play a role in the CV disease of RA patients.

Histological studies have studied the association of the complement system with CV disease. Specifically, the assessment involved measuring the quantity of C1q expression at different stages of atherosclerosis through techniques such as immunohistochemistry, western blotting, and real-time polymerase chain reaction, using abdominal aortas obtained from autopsy cases. Interestingly, C1q immunoreactivity was localized in the extracellular matrix, necrotic cores, macrophages and smooth muscle cells in atherosclerotic lesions, and western blotting and real-time polymerase chain reaction showed that C1q protein and mRNA expression was significantly higher in advanced lesions than in early lesions [29].

Genetic evidence has also linked the complement system and CV disease. For example, deficiency in the classical pathway component C2 predisposes individuals to an increased risk of myocardial infarction, potentially due to decreased classical pathway-mediated clearance of immune complexes [30]. Similarly, in a randomized, controlled trial, 12 single nucleotide polymorphisms in the *CR1* gene (which encodes complement receptor 1) were associated with the risk of incident coronary artery disease [31].

Based on our study findings and the heatmap representation we generated, it appears that the association of the complement system with cIMT and carotid plaque corresponds to components of the complement located in the common and terminal pathways, including downstream activated particles. This trend was also observed in the healthy population. In this regard, elevated plasma levels of C5 were found to be associated with subclinical atherosclerosis, plaque volume, and coronary calcification in two distinct cohorts [32]. Furthermore, increased plasma levels of C5a were linked to higher CV risk in patients with advanced atherosclerosis [33].

Distinct pathophysiological mechanisms contribute to the formation of carotid plaque and cIMT. Unlike cIMT, carotid plaque primarily consists of intimal thickening characterized by the presence of foam cells, smooth muscle cells, macrophages, a lipid core, and a fibrous cap, which vary depending on the stage of plaque development [34]. Given that both processes involve distinct pathophysiological mechanisms, it could be hypothesized that they also exhibit distinct patterns of association with the complement system. This fact may explain why the pattern of complement molecules associated with cIMT and carotid plaque displays in our study differences in certain aspects.

In our work, we observed numerous univariable relationships with several complement components. However, many of these associations were attenuated following multivariable analysis. The complement system has been closely linked to various cardiometabolic comorbidities, including insulin resistance, type 2 diabetes, dyslipidemia, obesity, and fatty liver disease [35]. However, although traditional CV risk factors may have acted as confounding variables in the relationship between the complement system and carotid evaluation, it is worth noting that despite this, the complement system maintained significant associations with both cIMT and with carotid plaque in the multivariate analysis.

We acknowledge several limitations in our study. In this regard, it was cross-sectional, so causality cannot be inferred. Besides, the complement system is a dynamic cascade with multiple regulators and inhibitors, so a static picture may not accurately represent its true pathophysiology. However, it has several strengths. Specifically, it involved a comprehensive assessment of a wide range of complement molecules along with detailed phenotyping of study participants, facilitating thorough adjustment for potential confounders. Furthermore, none of the previously discussed studies were conducted in inflammatory conditions such as RA, nor did they include as comprehensive analysis of the complement system as ours. We also acknowledge that the different types of therapies used for the disease might have affected complement system values even though some of them were included in the multivariable analysis. Prospective studies will be needed to evaluate the effect of treatments on the complement system in patients with RA.

## Conclusion

In conclusion, the complement system, especially its activated elements from the common and terminal pathways, is independently and positively associated with arteriosclerotic disease in patients with RA.

### Electronic supplementary material

Below is the link to the electronic supplementary material.

Supplementary Material 1

## Data Availability

The data sets used and/or analyzed in the present study are available from the corresponding author upon request.

## Abbreviations

ACPA: Anti-citrullinated protein antibodies
BMI: Body mass index
CDAI: Clinical Disease Activity Index
cIMT: Carotid intima media thickness
CRP: C reactive protein
CV: Cardiovascular
DAS28: Disease Activity Score in 28 joints
DMARD: Disease-modifying antirheumatic drug
ESR: Erythrocyte sedimentation
HDL: High density lipoprotein
LDL: Low density lipoprotein
NSAID: Nonsteroidal anti-inflammatory drugs
RA: Rheumatoid arthritis
SDAI: Simple Disease Activity Index
TNF: Tumor necrosis factor, rate
