# Supplementary material for: The Relationship between the complement system and subclinical carotid atherosclerosis in patients with rheumatoid arthritis
Source: Arthritis Res Ther. 2024 Jul 8;26:127. doi: 10.1186/s13075-024-03360-3 (PMC11229295; doi:10.1186/s13075-024-03360-3)
Supplement: Supplementary file 1 — Supplementary Material 1 [file 13075_2024_3360_MOESM1_ESM.docx]

| **Supplementary Table 1. Functional test and individual elements values of the complement system in RA patients.** | | | |  |
| --- | --- | --- | --- | --- |
| Functional complement assays, % | | | |  |
| Classical pathway | | 96 ± 24 | |  |
| Alternative pathway | | 90 ± 26 | |  |
| Lectin pathway | | 50 (7-106) | |  |
| Individual complement components | | | |  |
| Classical pathway | |  | |  |
|  | C1q, mg/dl | 33 ± 8 | |  |
| Alternative pathway | |  | |  |
|  | Factor D, mg/dl | 0.17 ± 0.07 | |  |
|  | Properdin, mg/dl | 1.3 ± 0.4 | |  |
| Lectin pathway | |  | |  |
|  | Lectin, mg/dl | 0.08 (0.03-0.19) | |  |
| Classical and lectin pathways | | |  | |
|  | C1-inhibitor, mg/dl | 32 ± 7 | |  |
|  | C2, mg/dl | 7 (4-11) | |  |
|  | C4, mg/dl | 27 ± 10 | |  |
|  | C4b, mg/dl | 6 ± 3 | |  |
| Common pathway | |  | |  |
|  | C3, mg/dl | 141 ± 29 | |  |
|  | C3a, mg/dl | 34 ± 10 | |  |
|  | Factor I, mg/dl | 4 ± 1 | |  |
| Terminal pathway | |  | |  |
|  | C5, mg/dl | 3.9 ± 1.9 | |  |
|  | C5a, mg/dl | 1.0 (0.7-1.4) | |  |
|  | C9, mg/dl | 1.0 (0.6-1.3) | |  |

Data represent mean ± SD or median (IQR) when data were not normally distributed.

486 RA patients preselected from out-patients clinic

n=465

n=430

RA patients meeting all inclusion and exclusion criteria

n=12 Other chronic disease

n=8 Cardiovascular events

n=1 Active infection

n=35

Prednisone equivalent dose > 10 mg

**Supplementary Figure 1.** Flowchart of excluded and included patients.
